# Antitoxin in Membranous Croup

**Published:** 1897-10

**Authors:** R. E. L. Barnum

**Affiliations:** Richland, Ga.


### ANTITOXIN IN MEMBRANOUS CROUP.

RICHLAND, GA., August 26, 1897.

*Atlanta Medical and Surgical Journal:*

On August 11th, at about 8 p.m., my partner, Dr. R. T. Crozier, was called to see a negro child, female, two years and eight months of age, whom he found suffering with membranous croup. An emetic of ipecac and alum was prescribed, and followed by calomel and soda, two grains each, every two hours, until three doses were taken. Also tr. ferri. chlor., chlorat. potass. and glycerine. Throat wash of aqua calcis and carbolic acid.

The first manifestations of the disease had taken place the previous night, and at this time, twenty-four hours later, there was evidence of considerable obstruction and loud noisy respiration. On the following morning I visited the case with him and we found all the symptoms of the previous night intensified. The dyspnea was very great. The suprasternal, inframammary, post-clavicular and epigastric regions were markedly depressed at each effort at inspiration. The abdominal muscles were also brought into play. Temperature 100 degrees Fahrenheit, pulse 140. On examination of throat we found almost the entire pharynx covered with a yellow dirty looking deposit, including the tonsils, palatine arches and uvula, which latter was almost completely encircled by the pseudo-membrane, the exception being a small space extending down its anterior surface, where the membrane failed to meet. There was no enlargement of any of the glands, and very little excretion of saliva or mucus, rendering the cough dry and harsh. At this time another emetic of ipecac and alum was administered, with but little signs of relief. As the symptoms were rapidly growing worse, and we saw no hope for our patient under the treatment being used, we decided to test the virtues of antitoxin. Accordingly, at 11:30 a.m. of the 12th, we inserted 1000 units of the serum, using Parke, Davis & Co.'s product. An ordinary hypodermic syringe was employed, having been first thoroughly cleansed and sterilized. Being necessary to make several injections, we decided to make them at different places on the body, selecting right and left hypochondriac regions and flexor surface of thighs. It was rapidly taken up, and no abscess resulted from either insertion. Former treatment was continued. At our next visit, at 5 p.m., about five or six hours after injection, we found no change in symptoms; treatment continued. Visited patient again at 8:30 p.m., condition same, respiration still labored and noisy, patient beginning to show signs of exhaustion. Treatment was continued, had but little expectation of seeing patient alive next morning. At 9 a.m., 13th, found patient's condition improved, was told that change took place about midnight, or about twelve hours after injection of serum. Respiration still labored, but less noisy, cough somewhat looser. Bowels had not moved in twenty-four hours; gave five grains each calomel and soda. Examination of throat showed considerable quantity of membrane had disappeared, leaving but few patches here and there. Continued same treatment. At 5 p.m. respiration not so labored and almost noiseless, but still evidence of considerable obstruction. Calomel had acted, large lump of tenacious yellow substance passed, which was supposed to be cast-off membrane which child had swallowed. After fits of coughing it would always swallow, never spitting out anything. At 8 a.m., 14th, found patient greatly improved, respiration noiseless, free and easy. Temperature 99.6 degrees. Throat showed only a few small deposits of pseudo-membrane left. Patient continued to improve, and was dismissed on the morning of the 15th, throat entirely free of all deposits, temperature normal, appetite good, bowels and kidneys acting freely.

Alcohol was given throughout the disease in teaspoonful doses every two hours. Had this case occurred in a locality where diphtheria is prevalent it would doubtless have been diagnosed as such, but from a residence in this place of over ten years I have never known a case to occur. Every case of membranous croup that has come to my knowledge was directly traceable to exposure, this child having played in water up to its knees, during a shower a day or so previous to manifestations of the disease. No other case had occurred in the family previous to this one, nor subsequently. Besides, the general characteristics of the disease are sufficient to distinguish it from diphtheria. The absence of sequelæ, the lack of faucal swelling and glandular enlargement, the small quantity of salivary secretion, the absence of putridity, all point to membranous croup, a local and non-contagious disease.

I report this case because it is the first one that has come to my knowledge where antitoxin has been used with a successful result.

I intend to give it further trial.

R. E. L. BARNUM, M.D.

